# Editorial: Multifunctionalized smart materials and nanocarrier for medical imaging and drug delivery

**DOI:** 10.3389/fbioe.2024.1440354

**Published:** 2024-06-20

**Authors:** Xuan Zhou, Kuikun Yang, Cheng Gao, Kunchi Zhang

**Affiliations:** ^1^ Department of Mechanical and Aerospace Engineering, George Washington University, Washington, DC, United States; ^2^ Harbin Institute of Technology, Harbin, Heilongjiang, China; ^3^ Department of Pharmaceutical Sciences, University of Macau, Taipa, China; ^4^ Shanghai University of Medicine and Health Sciences, Shanghai, China

**Keywords:** biomedicine, nanomaterial, targeted drug delivery, bioprinting, biomaterial

Biomedicine brings huge prospect and potential for clinic patients and medical care system. All the achievements and efforts are for improving quality of life and conquering the threat of diseases. The rapid advancement of science has made a variety of traditional medical treatments increasingly changed. A growing number of novel materials, methods and technologies are involved to promote the development of biomedicine. Multifunctionalized materials were fabricated into varied nanocarrier for biomedicine applications. To address the focus, many researchers contributed their promising achievements to our Research Topic.

Targeted drug delivery is essential for precise cancer treatment. Yuan et al. designed a sequentially triggered strategy based on double targeting elements to release the drug on cancer site. The results demonstrated that the DNA nanocapsule had a specific inhibition effect on target cell metastasis. The strategy could be provide deep sight into the next-generation of targeted drug delivery.

Nanoparticles (NPs)-based immunotherapy had been utilized for decades. Li et al. prepared polyethylenimine (PEI) hybrid thin shell hollow mesoporous silica NPs (THMSNs) to load photosensitizer chlorine e6 (Ce6) for realizing the synergy of photodynamic therapy (PDT)/immunotherapy. The results implied that THMSNs-mediated and PDT-triggered nontherapeutic system with immunogenic property is a promising immunotherapy for cancer clinical treatment.

Three dimensional bioprinting technology plays a crucial role in biomedicine by fabricating artificial tissue or organ *in vitro*. Ma et al. printed a complex scaffold by stereoscopic projection lithography technology. The scaffold displayed multilayered micro structures to support stem cell growth and proliferation. The results demonstrated that three dimensional bioprinting technology show great prospects in future tissue engineering application.

Nanocomposite hydrogels had been widely used for serving carriers of drug delivery systems. However, bibliometric and visualized analysis are not common in this area. Wang et al. identified and retrieved the publication concerning the application of NIH for drug delivery in past 20 years. The resultant of bibliometric and visualized offered an investigative study and comprehensive understanding of publications regarding applications of Nanocomposite hydrogels.

Traditional cancer therapies, including surgery, radiotherapy, chemotherapy, immunotherapy, and hormone therapy, are not satisfied to cure various cancer diseases. Nanomaterials for targeted drug delivery and nucleic acid aptamers bring new sights for targeted cancer therapy due to their high stability, high affinity, and high selectivity. Yin et al. introduced the characteristics of aptamer and nanomaterials, and the advantages of aptamer-functionalized nanomaterials (AFNs) in cancer treatments. Furthermore, the progress and challenges of AFNs were also discussed in this review.

